# Striatal Infusion of Glial Conditioned Medium Diminishes Huntingtin Pathology in R6/1 Mice

**DOI:** 10.1371/journal.pone.0073120

**Published:** 2013-09-12

**Authors:** Juan Perucho, Maria José Casarejos, Ana Gómez, Carolina Ruíz, Maria Ángeles Fernández-Estevez, Maria Paz Muñoz, Justo García de Yébenes, Maria Ángeles Mena

**Affiliations:** 1 Department of Neurobiology, Hospital “Ramón y Cajal”, Madrid, Spain; 2 Department of Neurology, Hospital “Ramón y Cajal”, Madrid, Spain; 3 CIBERNED, Madrid, Spain; University of Florida, United States of America

## Abstract

Huntington's disease is a neurodegenerative disorder caused by an expansion of CAG repeats in the huntingtin gene which produces widespread neuronal and glial pathology. We here investigated the possible therapeutic role of glia or glial products in Huntington's disease using striatal glial conditioned medium (GCM) from fetus mice (E16) continuously infused for 15 and 30 days with osmotic minipumps into the left striatum of R6/1 mice. Animals infused with GCM had significantly less huntingtin inclusions in the ipsilateral cerebral cortex and in the ipsilateral and contralateral striata than mice infused with cerebrospinal fluid. The numbers of DARPP-32 and TH positive neurons were also greater in the ipsilateral but not contralateral striata and substantia nigra, respectively, suggesting a neuroprotective effect of GCM on efferent striatal and nigro-striatal dopamine neurons. GCM increases activity of the autophagic pathway, as shown by the reduction of autophagic substrate, p-62, and the augmentation of LC3 II, Beclin-1 and LAMP-2 protein levels, direct markers of autophagy, in GCM infused mice. GCM also increases BDNF levels. These results suggest that CGM should be further explored as a putative neuroprotective agent in Huntington's disease.

## Introduction

Huntington's disease (HD) is an inherited neurodegenerative disorder as a result of the expansion of a CAG trinucleotide repeat in the huntingtin gene (HTT). Expanded huntingtin protein (htt) produces neuronal loss wich is more pronounced in the striatum, involving preferentially striatal efferent small and medium spiny neurons [1], [2]. Cognitive deficits and parkinsonian symptoms are frequent in patients with HD, suggesting involvement of other brain regions such as the cerebral cortex and the nigrostriatal pathways that have been found altered in neuropathological studies [2].

There are few studies concerning the role of glia in HD. There is glial pathology in human patients with HD [3], [4], [5] and in animal models of this disease [6], [7]. Mutant HTT is also expressed in glial cells and affects directly the neuron pathology in HD [8]. Htt inclusions, however, are much more common in neurons than in glia in mutant mouse brains [9], [10], suggesting that glial cells have mechanisms that prevent the accumulation and deposition of htt aggregates in glia.

R6 mice are a model of HD developed by Mangiarini [11] and have been the most used. One of the lines, the R6/1, has a CAG expansion of approximately 115. These animals develop pathology around 15–21 weeks and die around 32–40 weeks [12], [13]. They display nuclear and cytoplasmic abnormalities in the corpus striatum and hippocampus [14], [15], [16], reduction in dopamine D1 and D2 receptor binding and DARPP-32 levels in striatum [17], [18].

Glia conditioned medium (GCM) is the result of culturing glia for 24 hours in defined culture medium. GCM is rich in antioxidants and neurotrophic factors and it has been found to be neuroprotective in some models of neurodegenerative diseases [19], [20], [21], [22]. We have shown than in Q7 and Q111 striatal neurons, a cellular model of polyglutamine expansion, GCM has a neuroprotective effect [23]. In this study we have tested the neuroprotective effects of GCM *in vivo* on the R6/1 model of HD.

## Methods

### Ethics statement

All procedures used in this work were in accordance with the European Union Council Directive (86/609/EEC). The protocol was approved by the Committee on the Ethics of Animal Experiments of the Hospital "Ramón y Cajal (animal facilities ES280790002001).

### R6/1 mutant mice and genotype determination

#### HD mutant mice

R6/1 male founders, expressing exon 1 of the human huntingtin gene with 115 CAG repeats were donated by Prof. Lucas [11]. All efforts were made to minimize the number of animals and their suffering. Twenty-four male mice were used, 6 mice for each group in the two different experimental treatments (15 and 30 days of infusion). The 15 day treated mice were used for biochemical and the 30 day treated mice for histological analysis. All the animals were implanted with the Alzet pump at 3 months and 15 days of age and were sacrificed at 4 months in the first experiments, and at 4 months and 15 days in the second experiment, by cervical dislocation.

#### Genotype determination

Genomic DNA was extracted from mouse tail after proteinase K digestion (16 h at 55°C) in lysis buffer according to the manufacturer's instructions (High Pure PCR template preparation kit, Roche, Barcelona, Spain). For genotyping R6/1 mice 150 ng of genomic DNA were denatured for 3 min at 94°C and subjected to 35 cycles of 1 min at 94°C, 1 min at 55°C, and 1min at 72°C, followed by 5 min of a final extension at 72°C. PCRwas performed in a final volume of 25 μL containing 0.75 U of Taq DNA polymerase (Promega, Madrid, Spain; 5 U/μL), 0.2 mM dNTP, 1.5 mM MgCl2, 5 mM TrisHCl (pH 8.0), 0.5 μL of DMSO and 1.5 μL of specific sense and antisense primers at 10 μM. The specific primers were:

HD-1D: 5′-CCGCTCAGGTTCTGCTGCTTTTA-3′.

HD-1R: 5′-TGGAAGGACTTGAGGGACTC-3′.

Twenty microlitres of the PCR reaction products were analyzed by electrophoresis in a 1.8% agarose gel that was subsequently stained with GelStar® from Cambrex (Bio Science Rockland, Inc.) for visualization of DNA bands. DNA molecular weight markers (Roche, Spain) were used to provide a size reference for the test reactions. The sizes of PCR products are used for the identification of the genotype, 170 bp for mutant huntingtin.

### Glial cultures and striatum glia-conditioned medium (GCM)

#### Culture media and reagents

DMEM with high glucose (4.5 g/L), Ham's F-12 nutrient mixture, Eagle's minimal essential medium (EMEM) with Earl's salts, Leibovitz's L-15 medium, B27/Neurobasal TM medium, HBSS, L-glutamine, pyruvate, penicillin, streptomycin and fetal bovine serum (United States origin) were purchased from Invitrogen (Carlsbad, CA). Glucose at 45%, trypsin–EDTA, insulin, putrescine, progesterone, sodium selenite, and poly-D-lysine were from Sigma (Madrid, Spain), and human transferrin, 30% iron-saturated, was from Roche Diagnostics (Barcelona, Spain).

All other agents were of the highest commercially available purity, from Merck (Darmstadt, Germany) or Sigma. The radiochemical [3H]DA (70 Ci/mmol) was obtained from DuPont NEN (Boston, MA).

#### Cell cultures

Glial striatum cultures E16 (16^th^ day of gestation) and midbrain neuronal-enriched primary cultures E13 (13^th^ day of gestation) were obtained from C57BL/6 wild-type (WT) mice. All efforts were made to minimize the number of animals used and their suffering.

**For Glial cultures**, the striatum was removed from embryonic tissue E16, diced in small fragments, and incubated in trypsin-EDTA (0.5% in HBSS) at 37°C for 15 min. Trypsinization was stopped by adding culture medium, and the tissue was gently centrifuged. The supernatant was discarded, and the pellet was resuspended in 1 ml of culture medium. Single-cell dissociation was achieved by mechanical disruption. Dissociated cells were plated in DMEM with 15% (v/v) heat-inactivated fetal bovine serum, 4 mM L-glutamine, 1 mM pyruvate, and 100 U/ml penicillin–streptomycin (growth medium; DMEM–FCS) [19] at a density of 3×10^6^ cells per 80 cm^2^ cell culture flask. Culture medium was refreshed after 6–7 days and every 7 days thereafter. After 18 d in culture, positive staining with anti GFAP antibody identified the astrocytes in these cultures and comprised at least 80–90% of total cells.

#### Glía Conditioned Medium

In order to obtain the GCM, DMEM-FCS medium was discarded, the cells were washed three times with Leibovitz's L-15 medium and subsequently cultured in a chemically serum-free defined medium (EF12) [24], [25]. After 24 h of culture under such conditions, the medium was collected and stored frozen. This medium was considered GCM. EF12 consisted of a 1∶1 (v/v) EMEM and nutrient mixture of Ham's F-12, supplemented with D-glucose (6 mg/ml), insulin (25 mg/ml), transferrin (100 mg/ml), putrescine (60 mM), progesterone (20 nM), and sodium selenite (30 nM).

#### Neuronal midbrain primary cultures

Neuronal-enriched midbrain primary cultures were obtained and prepared as described previously [26]. The ventral midbrain was removed from embryonic tissue (E13) and incubated with 0.36 mg/ml papain in phosphate buffered saline (PBS) solution (2.7 mM KCl, pH 7.4, 137 mM NaCl, 10 mM Na_2_HPO_4_)/D-glucose (6 mg/ml)/1% BSA buffer for 15 min at 37°C and mechanically dissociated in the presence of 10 mg/ml DNase-I. The cells were seeded in B27/Neurobasal TM medium with 15%(v/v) heat-inactivated fetal calf serum (B27/NBL–FCS) supplemented with glutamine (4 mM) and penicillin-streptomycin (100 U/ml) at a density of 2.5×10^5^ cells/cm^2^ in multiwells or 2×10^5^ cells/cm^2^ in glass cover slides precoated with poly-D-lysine (4.5 µg/cm^2^) in 0.1 M borate buffer, pH 8.4, and laminin (3 µg/ml). The cultures were kept in a humidified chamber at 37°C in a 5% CO_2_ atmosphere for 7–8 days *in vitro*. Twenty-four hours after plating, the cells were changed to serum-free medium (B27/NBL).

#### ^3^H-Dopamine uptake

[^3^H] DA uptake was measured after incubation of these 7–8 DIV neuronal midbrain primary cultures (E13) with 10^−8^ M [^3^H] DA (70 Ci/mmol), in the presence of 10^−5^ M pargyline, and 10^−3^ M ascorbic acid, at 37°C for 20vmin. Nonspecific uptake/binding was calculated in the presence of 1–10 µM GBR 12935 (dopamine transporter inhibitor) and represented ≤5% [24], [25].

#### Artificial Cerebro Spinal Fluid

Artificial cerebrospinal fluid (CSF) for infusion mirrors the composition of endogenous cerebrospinal fluid, making it therefore physiologically compatible. Artificial CSF was prepared by mixing two components, A and B, in 1∶1 proportion. Component A: 148 mM NaCl, 3 mM KCl, 1.5 mM CaCl_2_ and 1 mM MgCl_2_. Component B was composed of 0.8 mM Na_2_HPO_4_ and 0.2 mM NaH_2_PO_4_.

### Intra-striatum infusion

**Figure 1:**
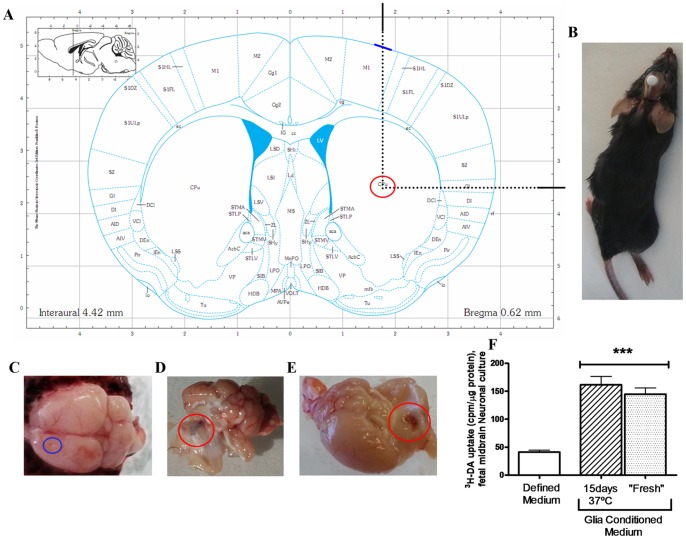
Method and implantation coordinates into striatum. (**A**) Paxinos coordinates chosen for striatal infusion in mice aiming the centre of the left striatum at AP: +0.62 mm; ML: −1.75 mm to bregma a v d DV: −3.5 mm to the skull. The injection rate was 0.25 μl/hour for both experimental times. (**B**) Image of the implantation point in the mouse brain. (**C, D**) Methylene blue staining in the striatum, after 15 and 30 days of pump implantation. (**E**) HD mouse with pump installed subQ on the back and the brain infusion kit fixed with methacrylate to the skull. (**F**) High-affinity [^3^H]DA uptake in GCM “fresh” (immediately thawed), GCM incubated at 37° C, for 15 days compared with Defined medium (DM). The values are expressed as the mean ± SEM (n = 5). Statistical analysis was performed by one-way ANOVA, followed by Newman–Keuls multiple comparison test. ***p<0.001 GCM *vs* DM.

Under Isoflurane anesthesia, R6/1 mice were implanted in the left hemibrain with a 28-gauge infusion cannula (brain infusion kit I; Alzet®) connected to an osmotic minipump, placed subcutaneously. The pumps used in these experiments were the model 1002 for 15-day infusion and, and the model 2004 for 30-day infusion. The 4.5-mm cannula was stereotaxically implanted aiming the centre of the left striatum at AP (Antero-Posterior): +0.62 mm; ML (Medium-Lateral): −1.75 mm with respect to bregma and DV (Dorso-ventral): −3.5 mm with respect to the skull (Figure 1A), according to the stereotaxic mouse brain atlas of Franklin and Paxinos (2007). The injection rate was 0.25 μl/hour for both experimental times. Before the implantation of the Brain Infusion Kit, the correct coordinates of the striatum were assured using methylene-blue as a marker in previous animals (Figure 1C-1E). Once the coordinates and methods were secure GCM and CSF were administrated into the left striatum through the Alzet© pumps.

The different times of the two experiments are due to a first 15-day approximation to study changes in mechanisms such autophagy or other effects produced by GCM. After these previous results, a longer experiment of 30-days was performed aiming to observe if there was a reduction in the accumulation of important proteins with the GCM infusion.

### Determination of monoamines and their metabolites

In order to determine monoamine levels and their metabolites after decapitation the hemi-brains were frozen on dry ice. The following monoamines were measured by HPLC with an ESA coulochem detector: Dopamine (DA) and its metabolites, 3,4-dihydroxyphenylacetic acid (DOPAC) and homovanillic acid (HVA); Noradrenaline (NA) and Serotonin (5-HT) and its metabolite, 5-hydroxy-indole-acetic acid (5-HIAA). Briefly, the tissue was sonicated in 6 vol. (weight/volume) of 0.4 N perchloric acid (PCA) with 0.5 mM Na_2_S_2_O_5_ and 2% EDTA and then centrifuged at 10,000×g at 4°C for 20 min. Monoamine levels were determined from 20 μl of the resulting supernatant. The chromatographic conditions were as follows: a column (Nucleosil 5C18); the mobile phase, a citrate/acetate buffer 0.1 M, pH 3.9 with 10% methanol, 1 mM EDTA, and 1.2 mM heptane sulfonic acid; and the detector voltage conditions: D1 (+0.05), D2 (−0.39), and the guard cell (+0.40), have been described previously [24].

### Histological studies

The animals were anesthetized intraperitoneally with a mixture (5∶4∶1) of ketamine (50 mg/ml), diazepam (1 mg/ml), and atropine (1 mg/ml) and perfused with 4% paraformaldehyde in PBS. After that, the whole brain was imbedded in paraffin, sectioned in the microtome at a thickness of 4 microns. We used rabbit polyclonal anti-dopamine and cAMP regulated neuronal phosphoprotein (DARPP-32) diluted 1/500 and Tyrosine hydroxylase (TH) from Chemicon (Temecula, CA, USA) diluted 1/2000. Mouse monoclonal anti-hungtintin (MAB5374) antibody diluted 1∶100 was from Chemicon (Madrid, Spain). We used a rabbit polyclonal anti-Lysosomal-associated membrane protein 2 (LAMP-2) antibody diluted 1∶500 from Abcam (Cambridge, UK). Anti-rabbit and anti-mouse secondary antibodies were from Dako (Denmark) diluted 1/100. The number of perinuclear immunoreactive LAMP-2 positive cells was counted in 6 striatum fields of each mouse at 20× using fluorescence microscope. DARPP-32 and huntingtin inclusions were counted in the striatum and cortex in both hemispheres, left and right, with a DAB-system (LSAB2 system, DACO) and visualized under optical microscopy.

The counting of cells was performed independently by two blind observers. In the SNpc, TH positive-cells were counted using a 10× objective. Images were taken with a Nikon Eclipse Ti microscope equipped with a Nikon DS-2MV camera using the scan large image function of Nikon NIS elements software to stitch widefield images encompassing the entire SN. The area occupied by the SNpc was delimitated and TH-positive cell counts were expressed as TH-positive cells/mm^2^.

### Protein analysis

The left and right hemibrains were sonicated (VibraCell, level 2 for 30 seconds) in six volumes (W/V) of 0.4 N perchloric acid with 0.5 mM Na_2_S_2_O_5_ and 2% EDTA and then centrifuged at 12000 rpm for 20 min at 4°C. The pellet, with the proteins, was neutralized (W/V = 1/6) with the lysis buffer (0.75% Na2CO3, 2% SDS, 0.25 mM PMSF, 10 mg/ml leupeptin, 2 mg/ml aprotinin, 10 mg/ml pepsin) and then sonicated and centrifuged at 13400 g for 30 min at 4°C. The supernatant was used for protein determination by BCA assay and for electrophoresis analysis. Samples (20–50 μg) were added to SDS sample loading buffer 2X (10% glycerol, 2% SDS, 0.1% bromophenol blue, 50 mM Tris, pH 6.8 and 5% β-mercaptoethanol), electrophoresed in 10% SDS-polyacrylamide gels and then electroblotted to 0.45 μm nitrocellulose membranes.

For immunolabeling, the blots were blocked with PBS and 5% dry skimmed milk for 1 h at room temperature. After blocking non-specific binding, the membranes were incubated overnight with specific antibodies in blocking solution at 4°C. Later, blots were washed twice with blocking solution for 10 min followed by another two washes with PBS for 5 minutes each. The blots were developed by chemiluminiscence detection using an odyssey assay with specific secondary IRDye (800CW and 680LT) antibodies from LICOR (Bonsai technologies) and quantified by computer-assisted video densitometry. β-actin was used as a loading control.

We used the following antibodies: rabbit polyclonal anti-DARPP-32 (1/1500), goat polyclonal anti-p62 (SQSTM-1) (1∶700) and rabbit anti-BDNF (1/1000) from Santa Cruz (Temecula, California). Rabbit polyclonal anti-beclin-1 (1∶1000) and mouse monoclonal anti-β-actin antibody diluted 1∶10000 were from Sigma. Mouse anti-HSC70 diluted 1/2000 and rabbit anti-LAMP-2A diluted 1/1000 were from Abcam (Cambridge, UK).

### Statistical analysis

The results were statistically evaluated with different tests. Significant differences between the experimental groups were analyzed with student's t test and with one-way ANOVA followed by Newman Keuls multiple comparison test. Differences were considered statistically significant when p<0.05.

## Results

### Glial Conditioned Medium is stable and neurotrophic in the pump infusion during 15 and 30 days of treatment

We performed some experiments in order to assure the correct placement of the infusion device and the stability of the glial conditioned medium. The target striatal area for implantation of the cannula is shown in figure 1 A, an anesthetized mouse with an implanted cannula is shown in Figure 1 B, and one brain with the tract of the cannula in the cerebral cortex is shown in Figure 1 C and 1 D.

There were no differences in the food and water ingestion or in the weight evolution between the animals infused with cerebrospinal fluid (CSF) or GCM. Behavioral tests before and during the experiment, including performance in the R-rod, activity in the A-track, and stride length (Figure S1) showed no differences between the different treatments.

To assure the stability of GCM during the period of infusion at body temperature, the GCM was kept 15 days at 37°C, and compared with recently thawed GCM. The GCM activity was tested *in vitro* midbrain neuronal cultures (E13) by 3H DA uptake. There were no differences between recently thawed and temperature incubated GCM (Figure 1 F).

### GCM treatment diminishes the number of huntingtin inclusions in cortex and striatum

**Figure 2:**
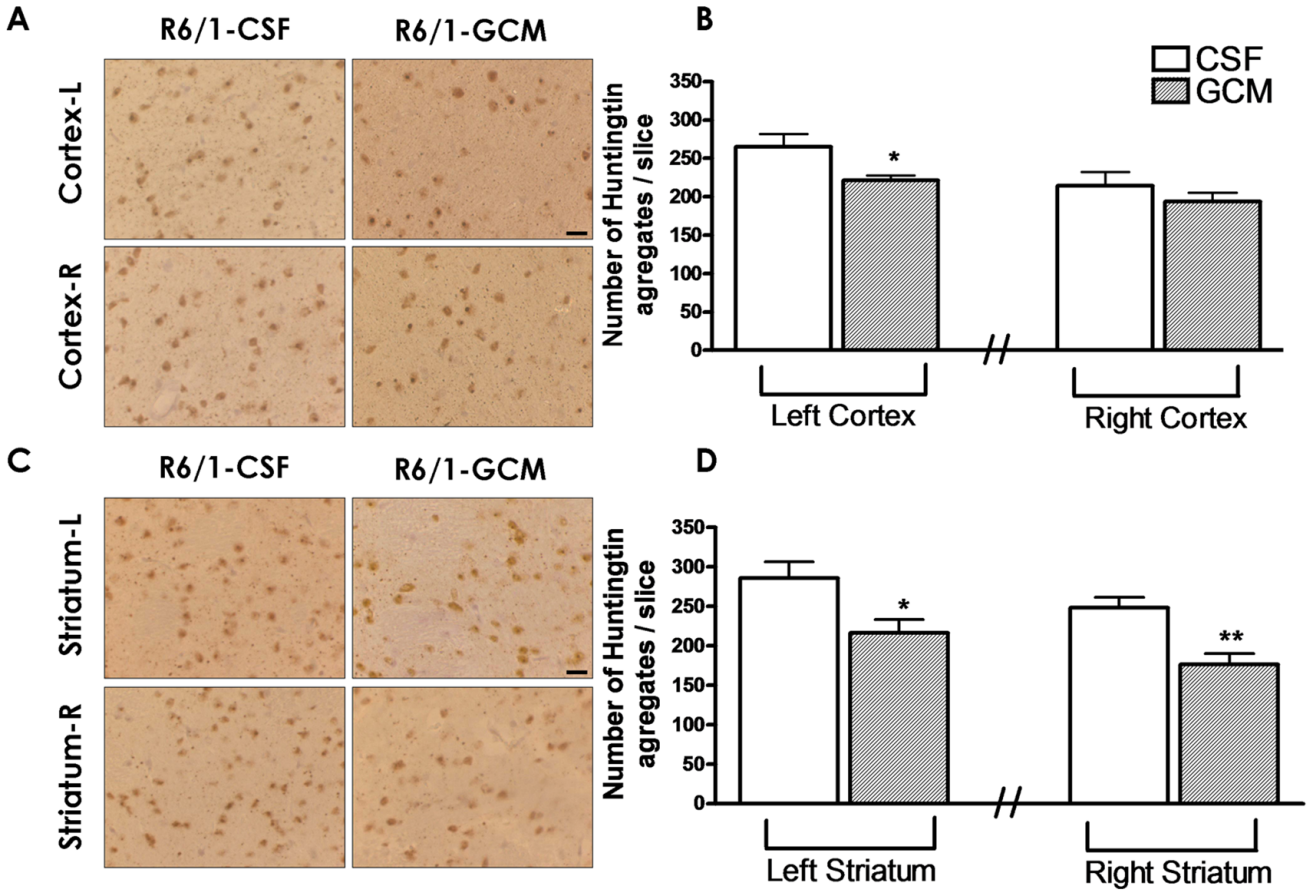
GCM treatment (30 days) diminishes the number of htt inclusions in cortex and striatum. (**A**) Microphotographs of the right and left cortex with the specific antibody anti-HTT inclusions and (**B**) quantification of both cortex, left and right. (**C**) Microphotographs of the right and left striatum with the specific antibody anti-HTT inclusions and (**D**) quantification of both striatum, left and right. The values are expressed as the mean ± SEM (n = 6 mice in each experimental group). The statistical analysis was performed by student's t test. *p<0.05, **p<0.01 GCM *vs* control CSF groups.

The number of huntingtin inclusions was reduced in the cerebral cortex and striatum of R6/1 mice infused with GCM (Figure 2). In the case of cortical inclusions, the differences were statistically significant in the ipsilateral cortex to the infusion but did not reach significance in the contralateral cortex to the infusion (Figures 2 A and B). In the case of the striatum, the reduction of huntingtin inclusions reached significant differences in both ipsilateral and contralateral striata (Figure 2 C and D).

### Glial Conditioned Medium treatment increases the number of DARPP-32 cells

**Figure 3:**
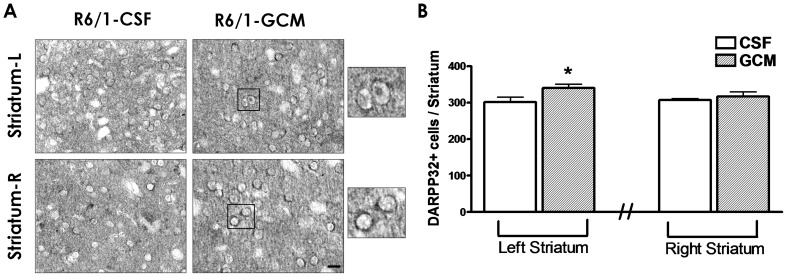
GCM treatment (30 days) increases the number of DARPP-32 cells. (**A**) Microphotographs of the left and right striatum with DARPP-32 positive cells and (**B**) quantification of the DARPP-32^+^ cells. A portion of the micrographs has been magnified to show cell DARPP-32 positive staining. The values are expressed as the mean ± SEM (n = 6 mice in each experimental group). The statistical analysis was performed by student's t test. *p<0.05 GCM *vs* control CSF groups.

DARPP-32 protein is a marker of striatal output neurons carrying dopamine 1 receptors, a subgroup of neurons heavily damaged in striatum of patients with HD and animal models of this disease. We found that the infusion of GCM increases the number of DARPP-32 positive neurons in the striatum ipsilateral to the infusion of GCM but not in the contralateral (Figure 3 A and B). This finding suggests that GCM partially prevents the decrease of striatal DARPP-32 positive neurons in HD.

### Glial Conditioned Medium protects the TH^+^ cells from substantia nigra pars compacta neurodegeneration in R6/1 mice

**Figure 4:**
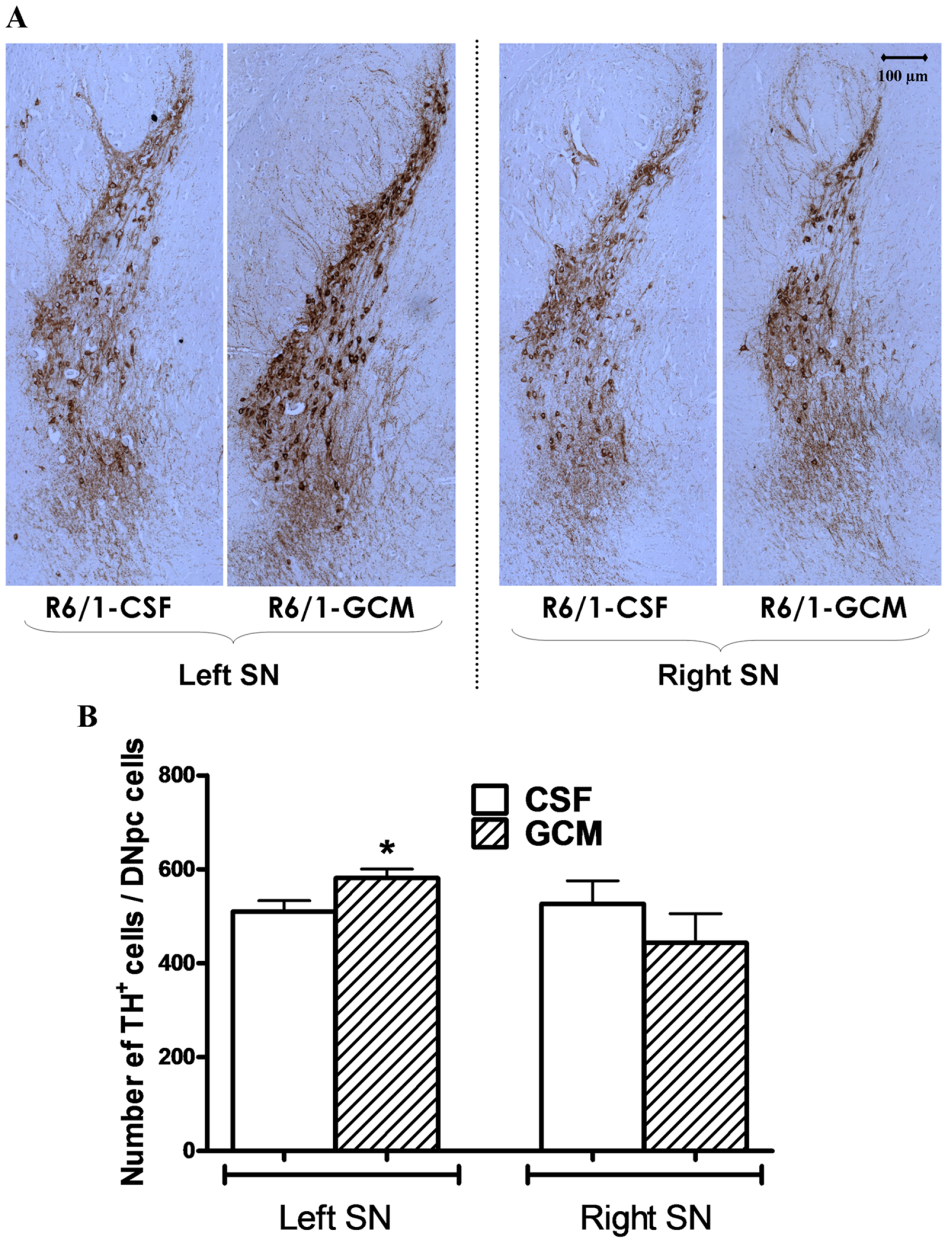
30^+^ cells in SNpc of the striatum *versus* CSF controls. (**A**) Stitched 10x images in order to encompass the whole Substantia Nigra Pars Compact (SNpc) in the striatum with TH positive cells (scale bar  = 100 µm). (**B**) Quantification of the number of TH cells positive cells/SNpc area. The values are expressed as the mean ± SEM (n = 3–6 mice in each experimental group). The statistical analysis was performed by student's t test. *p<0.05 GCM *vs* control CSF groups.

Symptoms of parkinsonism are present in most patients with HD and nigrostriatal dopamine neurons are damaged in patients with HD and in animal models of this disease. In these experiments, we found that the unilateral striatal infusion of GCM partially prevents the drop-out of tyrosine hydroxylase positive nigro-striatal neurons, without restoring the contralateral ones (Figure 4 A and B). This suggests that GCM or some of its active compounds are taken up by nigrostriatal dopamine terminals in the striatum and transported to the nigral neurons.

### Monoamine metabolism in 4-month-old R6/1 mice hemibrains after 15 days of GCM infusion

**Figure 5:**
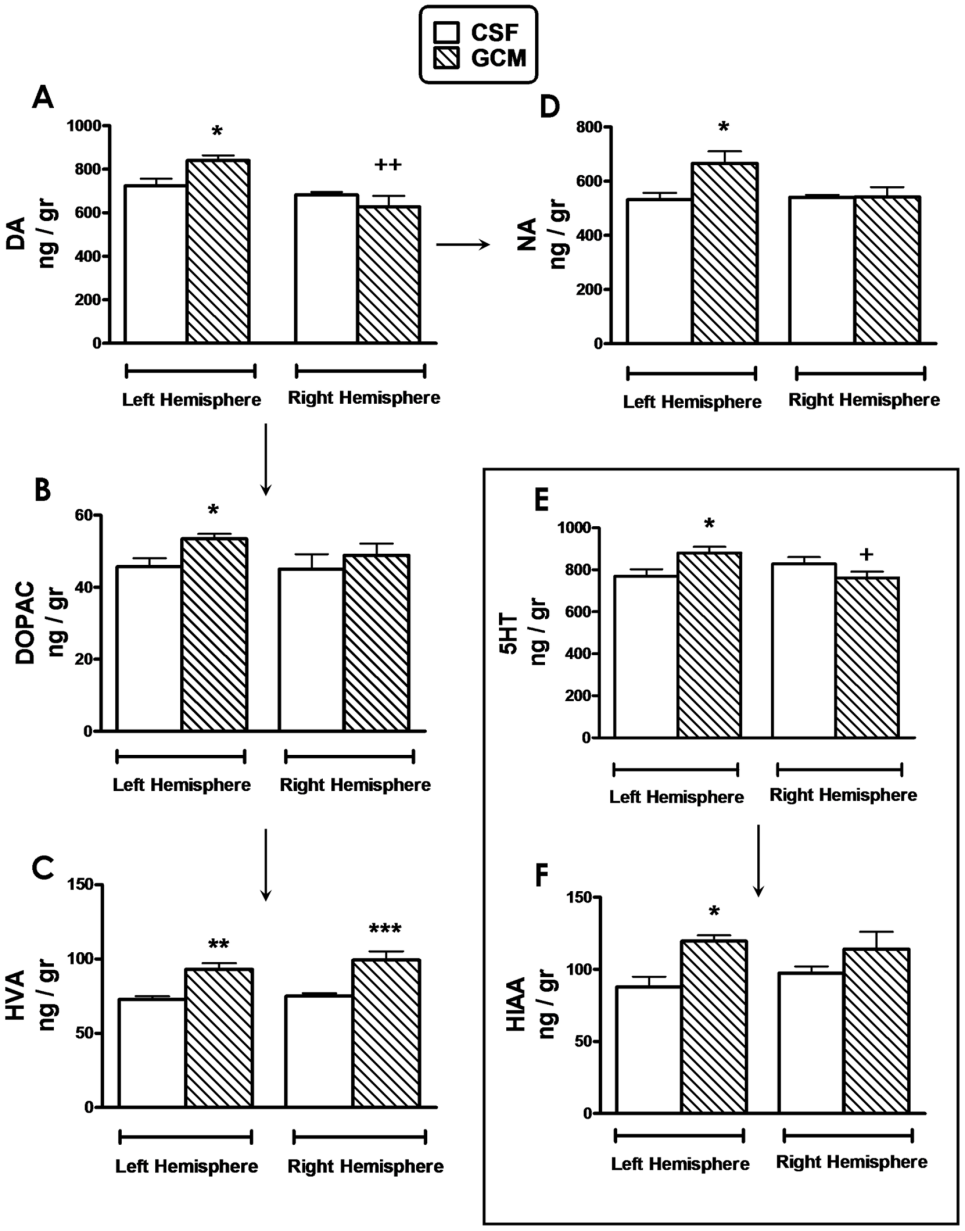
Monoamine metabolism in 4-month-old R6/1 mice hemibrains after 15 days of GCM infusion. (**A**) Dopamine, DA, (**B**) 3,4-Dihydroxyphenylacetic acid, DOPAC, (**C**) Homovalinic acid, HVA, (**D**) Noradrenaline, NA, (**E**) 5-hydroxytryptamine, 5HT and (**F**) 5-Hydroxyindoleacetic acid, HIAA measured by HPLC-ED. Results are expressed in nanograms per gram of fresh tissue (n = 6 mice per group). Statistical analysis was performed by student's t test. *p<0.05, **p<0.01 GCM *vs* control CSF groups; +p<0.05, ++p<0.01 right *vs* left hemispheres.

We found that the levels of dopamine and its metabolites were consistently increased in the ipsilateral cerebral hemibrain to the GCM infusion (Figure 5 A–C). Likewise, there was ipsilateral elevation of the levels of norepinephrine (Figure 5 D) and of serotonin and its metabolite 5-hydroxy-indole-acetic acid (5-HIAA, Figures 5 E–F). Changes in the contralateral hemibrain were inconsistent.

### Glial Conditioned Medium activates autophagy and increases BDNF levels

**Figure 6:**
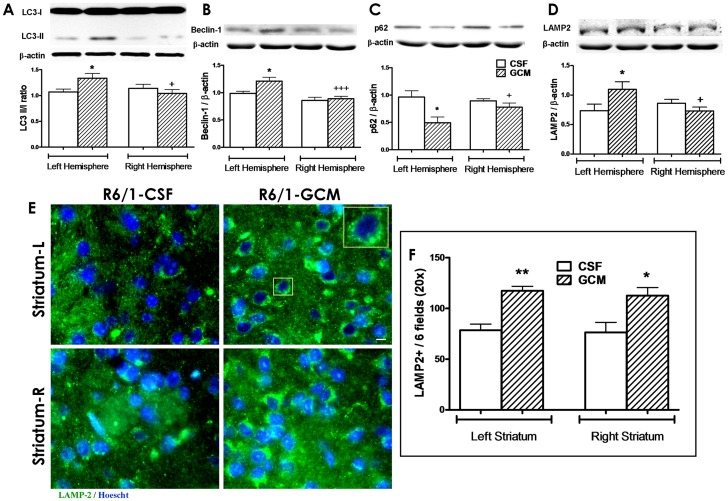
GCM treatment during 15 days activates autophagy pathways. (**A**) Representative bands and western blot quantification of LC3 II/LC3 I ratio. The presence of LC3 in autophagosomes and the conversion of LC3 to the lower migrating form LC3-II are used as an indicator of autophagy. (**B**) Representative bands and quantification of Beclin-1 antibody, direct marker of the autophagosome formation. (**C**) Western blots representative bands and quantification of p-62 specific antibody, as a substrate of autophagy and (**D**) LAMP-2 quantification, corrected by β-actin. Values are from the hemibrains, expressed as mean ± SEM (n = 6 in each group). Statistical analysis performed by t student. *p<0.05 GCM *vs* control CSF group and +p<0.05 right *vs* left hemispheres. (**E**) Representative microphotographs of Lysosomal-associated membrane protein, LAMP-2A antibody, present in lysosomes and endosomes, which implies autophagy activation. LAMP-2A staining (green) and nuclei (Hoescht, blue) immunofluorescence in striatum of R6/1 mice, with CSF and GCM infusion. (40× magnification, scale bar  = 10 µm). (**F**) Quantification of number of cells with LAMP-2A positive vesicle distribution in the perinuclear region, around the nucleus cell. Numbers of cells counted in 6 random fields of the striatum at 20× magnification. Mean ± SEM (n = 6 in each group). Statistical analysis performed by student's t test. *p<0.05, **p<0.01 GCM *vs* control CSF group; +p<0.05, +++p<0.001 right *vs* left hemispheres.

In this study, we found that infusion of GCM increased the levels of the markers of mature autophagy, such as LC3 II/I (Figure 6 A) and beclin-1 (Figure 6 B) and reduces the levels of the autophagy substrate p62 (Figure 6C) in the ipsilateral hemispheres. Likewise, GCM increases the levels of LAMP-2, a marker of activated autophagy (Figure 6 D–F).

**Figure 7:**
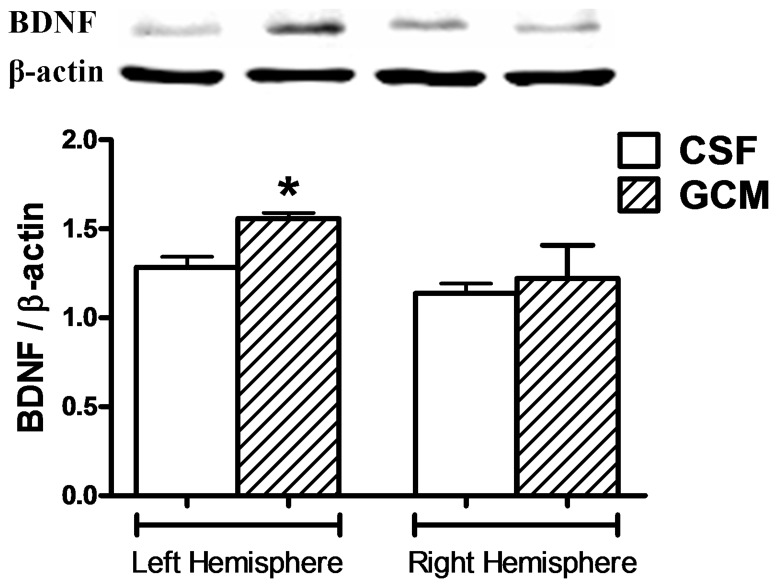
GCM treatment during 15 days increases brain BDNF. Western blot representative bands and quantification BDNF levels. Values are from the hemibrains, expressed as mean ± SEM (n = 6 in each group). Statistical analysis performed by student's t test. *p<0.05 GCM *vs* control CSF group.

In addition, the infusion of GCM for 15 days increases BDNF levels in the ipsilateral hemisphere (Figure 7).

## Discussion

In the present study, we demonstrate, in agreement with previous *in vitro* studies, a neuroprotective and positive effect of the GCM in a HD mouse model. We found that the GCM infusion into the striatum of R6/1 mice improves some of the abnormalities observed in this model, such as the presence of huntingtin inclusions in striatal and cortical neurons, the dropout of DARPP-32 immunoreactive efferent striatal cells and the loss of TH positive nigrostriatal neurons.

There is a gradient for the effects of GCM. Ipsilateral effects are more pronounced than contralateral. Reduction of huntingtin inclusions takes place in both striatum and cerebral cortex but more efficiently in the former, which is closer to the point of infusion, than in the latter. However, there is evidence for distant effects, such as the relative preservation of the nigro-striatal dopamine neurons, which suggests retrograde transport of the putative neuroprotective agents present in the GCM and infused in the striatum.

In spite of the improvement of the preservation of the mentioned neuronal systems in the mice infused with GCM, we did not find any significant improvement of behavior deficits observed in these animals. This lack of behavior effect is probably related to the restricted unilateral effect of the changes as well as to the small impact of a short term infusion, 15 or 30 days, in animals with genetic disorders progressing for several months.

The mechanisms of action of GCM are likely to be multiple. In previous studies in neuronal cultures *in vitro*, we have shown that GCM has powerful antioxidant properties. In this study, we have shown that GCM increases autophagy, which probably has a role in the elimination of huntingtin inclusions. The levels of BDNF provide a rationale for the increased preservation of both the striatal DARPP-32 immunoreactive and nigrostriatal TH positive dopamine neurons.

The role of glia in HD has not been thoroughly studied. Glial cells display nuclear htt aggregates but not as many as in neurons [9], [10]. Because of the htt, whether mutant or not, expression is ubiquous in the system, and the glial cells are also affected by the accumulation, however their role in neuronal degeneration and in the final HD pathology remains unclear. Glia and neuron are two cell lines in a very intimate union and very dependent on each other and their media. Due to this relation, both cell lines are influenced and affected by the same environmental stimuli. The mutant htt forms smaller aggregates in glial cells and in neurons shows earlier and more abundant formation of aggregates than in the glia cells. Furthermore, mutant htt in glial cells exacerbates neurological symptoms of Huntington disease mice [8].

The two major systems to remove misfolded proteins are the ubiquitin proteosomal system (UPS) and autophagy [27]. The glial cells could be able to clear the truncated proteins more efficiently than the neurons because of their different activity of the UPS, lower in neurons than in glia, but even lower in HD in almost all brain areas [28]. This could partially explain that misfolded polyQ proteins accumulate preferentially in the neurons [10]. Another possibility is an activation of autophagy in glia. Our results in p62, LAMP-2 and LC3 as autophagy markers point to this.

The involvement and the importance of the glia interaction in various neurodegenerative diseases [8], [9], [10] has been demonstrated [29], [30], [31]. The accumulation of mutant htt in astrocytes can exacerbate neurological symptoms in HD mice, there also results showing reduced BDNF levels and deficits in its axonal transport [32]. In addition, BDNF modulates dopaminergic deficits in a transgenic model of HD [32]. Astrocytes produce neurotrophic factors and cytokines to regulate the synaptic function and the neuron's morphology, and it has been tested that exacerbating the effect of htt glia produces an immediate effect in the HD pathology [33], [34]. Furthermore, BDNF regulates Rab11-mediated recycling endosome dynamics to induce dendritic branching and mutant huntingtin alters retrograde transport of TrkB receptors in striatal dendrites [35], [36]. In addition, the p75 neurotrophic receptor and BDNF can induce autophagy [37], [38]. Other growth factors, such as FGF, modulate autophagy by the PI3K/Akt pathway [39].

These factors secreted by the astrocytes could be stored and used as glial conditioned medium (GCM) in order to take advantage of their positive results. GCM is rich in small antioxidants, such as GSH and ascorbic acid, and peptidic neurotrophic factors [40], [41], [42], [43]. The GCM has a restorative effect in the dopamine cells in striatum, even after a 6-OH-dopamine infusion in the striatum [44]. This neuroprotective effect could be due to the antioxidant molecules, growth factors or the glial MAO-B action, which could reduce the amount of free radicals catabolizing the dopamine minimizing of the autoxidative effects of dopamine becoming neuromelanine [44]. *In vitro* studies using the GCM in Q111 cells, a striatal cell model of HD, showed promising results such as protection of degeneration after exposure to mitochondrial and oxidative stress insults [23].

Our results suggest that GCM could provide a new therapeutic strategy in HD, but several issues should be solved before GCM can be tested in human patients. The promising results found in R6/1 mice should be confirmed in models of HD in larger animals with larger brain volumes. Special delivery devices should be designed to distribute GCM in areas of the human brain, large enough to have an impact on the disease progression. Also we should explore whether human glia could be used to produce GCM as effective in patients with HD as that obtained from fetal rodent brain has been found in R6/1 mice. With these limitations, GCM is a promising new approach worthy of investigation in HD.

## Supporting Information

Figure S1 **Food, water intake, weight variation and behavior in the 30-days experimental group.** Quantity per mouse and day in food (grams) (**A**) and water intake (ml) (**B**) in the 30-days experiment. (**C**) Initial, pre-operation, and weight variation in the two experimental groups along the 30-days of experiment. (**D**) Table summarizing the data of the final behavior test, with Rota-rod, Acti-track and stride lenght at the end of the 30-days period, previous sacrifice. Values are expressed as mean ± SEM (n = 6 in each group).
(TIF)
