# Supplementary figures and images for: Striatal Infusion of Glial Conditioned Medium Diminishes Huntingtin Pathology in R6/1 Mice
Source: PLoS One. 2013 Sep 12;8(9):e73120. doi: 10.1371/journal.pone.0073120 (PMC3771920; doi:10.1371/journal.pone.0073120)

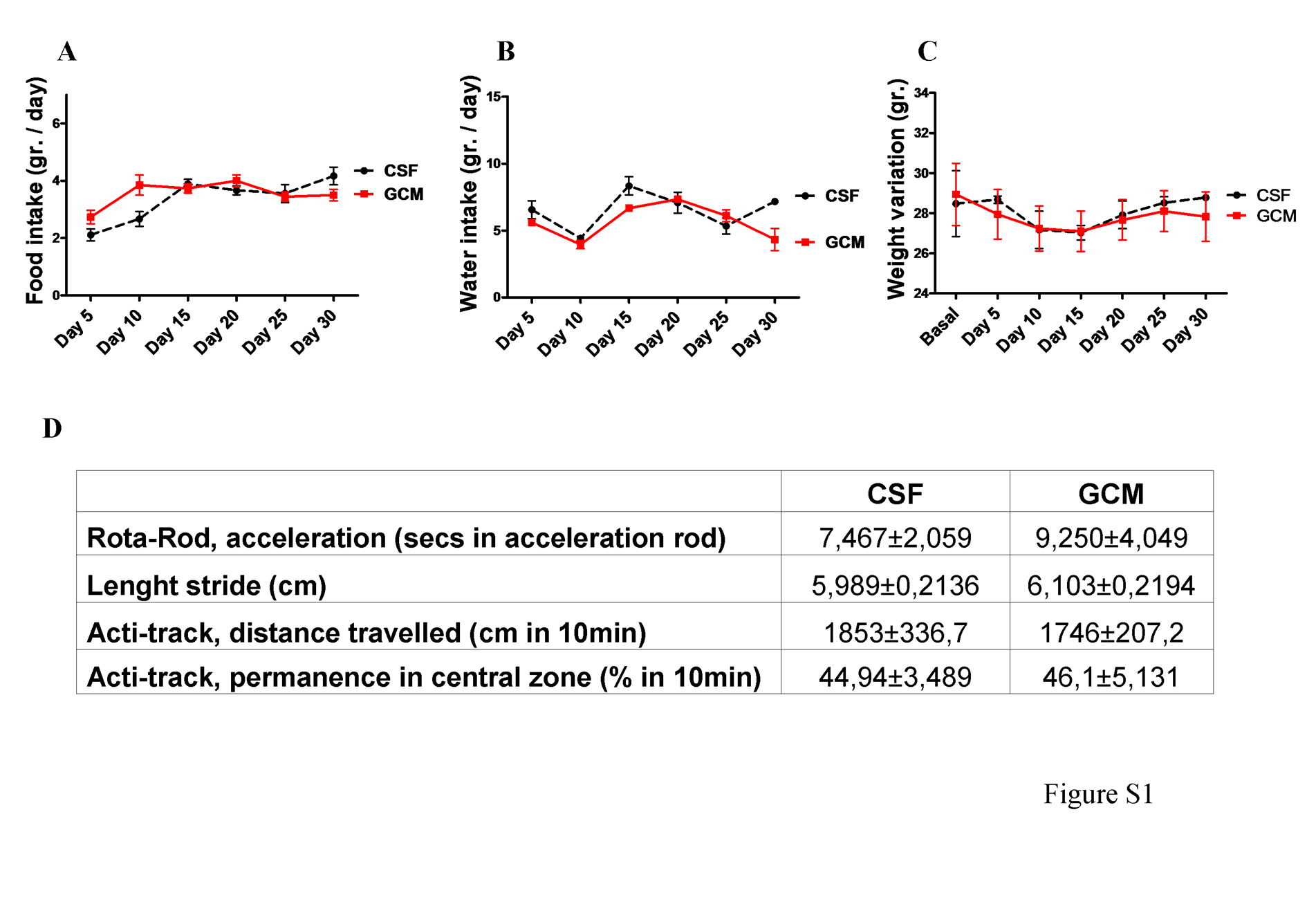

Supplement: Figure S1 — Food, water intake, weight variation and behavior in the 30-days experimental group. Quantity per mouse and day in food (grams) (A) and water intake (ml) (B) in the 30-days experiment. (C) Initial, pre-operation, and weight variation in the two experimental groups along the 30-days of experiment. (D) Table summarizing the data of the final behavior test, with Rota-rod, Acti-track and stride lenght at the end of the 30-days period, previous sacrifice. Values are expressed as mean ± SEM (n = 6 in each group). (TIF) [file pone.0073120.s001.tif]
